# InertDB as a generative AI-expanded resource of biologically inactive small molecules from PubChem

**DOI:** 10.1186/s13321-025-00999-1

**Published:** 2025-04-10

**Authors:** Seungchan An, Yeonjin Lee, Junpyo Gong, Seokyoung Hwang, In Guk Park, Jayhyun Cho, Min Ju Lee, Minkyu Kim, Yun Pyo Kang, Minsoo Noh

**Affiliations:** College of Pharmacy, Natural Products Research Institute, Seoul National University, Seoul, 08826 Republic of Korea

**Keywords:** Inactive compounds, Virtual screening, Synthetic negative data, Large-scale bioassay, Generative model, Predictive pharmacology

## Abstract

The development of robust artificial intelligence (AI)-driven predictive models relies on high-quality, diverse chemical datasets. However, the scarcity of negative data and a publication bias toward positive results often hinder accurate biological activity prediction. To address this challenge, we introduce InertDB, a comprehensive database comprising 3,205 curated inactive compounds (CICs) identified through rigorous review of over 4.6 million compound records in PubChem. CIC selection prioritized bioassay diversity, determined using natural language processing (NLP)-based clustering metrics, while ensuring minimal biological activity across all evaluated bioassays. Notably, 97.2% of CICs adhere to the Rule of Five, a proportion significantly higher than that of overall PubChem dataset. To further expand the chemical space, InertDB also features 64,368 generated inactive compounds (GICs) produced using a deep generative AI model trained on the CIC dataset. Compared to conventional approaches such as random sampling or property-matched decoys, InertDB significantly improves predictive AI performance, particularly for phenotypic activity prediction by providing reliable inactive compound sets.

**Scientific contributions**

InertDB addresses a critical gap in AI-driven drug discovery by providing a comprehensive repository of biologically inactive compounds, effectively resolving the scarcity of negative data that limits prediction accuracy and model reliability. By leveraging language model-based bioassay diversity metrics and generative AI, InertDB integrates rigorously curated inactive compounds with an expanded chemical space. InertDB serves as a valuable alternative to random sampling and decoy generation, offering improved training datasets and enhancing the accuracy of phenotypic pharmacological activity prediction.

**Supplementary Information:**

The online version contains supplementary material available at 10.1186/s13321-025-00999-1.

## Introduction

Predicting the biological activity and toxicity of chemical compounds for drug discovery has been revolutionized by artificial intelligence (AI) and the availability of extensive chemical datasets [1–5]. High-quality and sufficient bioactivity data on chemicals are crucial for developing accurate and reliable predictive AI models [6]. Bioassay databases like PubChem and ChEMBL, which compile bioactivity data for chemicals from high-throughput screening (HTS) assays and literature, have become indispensable resources for machine learning tasks in predictive modeling [7–9]. However, the application of these extensive datasets in AI-based predictive models for toxicology and pharmacology is often constrained by a lack of data on inactive compounds, i.e. negative data, and publication bias, as researchers predominantly report positive findings, skewing datasets towards biologically active compounds [10–12].

To address this bias and the deficit of data on inactive compounds, researchers commonly use random sampling from chemical databases such as PubChem [8], ChEMBL [9], or ZINC [13, 14]. This strategy supplements or replaces the insufficient data on inactive compounds with randomly sampled compounds, enhancing the robustness and accuracy of predictive AI models [15–19]. Additionally, AI-generated property-matched decoy sets, which include potential inactive compounds, have been employed [20], as demonstrated by datasets like DUD-E [21], DEKOIS [22], and the DeepCoy model [23]. Although these decoy chemical sets were initially proposed for structure-based virtual screening [24], such as molecular docking analysis, they have also been applied to various phenotypic pharmacological predictive models to incorporate inactive compounds in training datasets [25–28]. Currently, there are hardly any chemical databases for inactive or negative results constructed based on real activity data [12].

To fill this gap, we here introduce InertDB, a curated database designed as a comprehensive resource of biologically inactive small molecules, compiled from large-scale bioassay data. InertDB includes 3,205 inactive compounds, referred to as curated inactive compounds (CICs), identified through extensive curation of all available bioassay results in PubChem. Additionally, using deep generative AI model trained with the CICs, the chemical space of InertDB was expanded, resulting in 64,368 generated inactive compounds (GICs). InertDB, the first database enriched with negative data, provides a valuable resource for various chemical bioactivity predictive models, significantly enhancing the performance of AI models.

## Results

### Selection of CICs

**Fig. 1:**
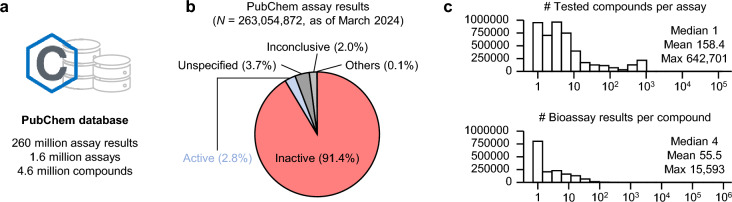
Statistics of PubChem database. **a**, Overview of PubChem Bioassay. **b**. Annotation for assay results in PubChem database. **c**. Histograms describing the number of tested compounds per assay and the number of available bioassay results per compound in PubChem database

To construct a comprehensive dataset of biologically inactive small molecules, we analyzed over 260 million assay results from PubChem, the largest available database for chemical bioactivity data [8, 29] (Fig. 1a). Each assay result was initially categorized as active, inactive, unspecified, or inconclusive (Fig. 1b). The majority of assay results were clearly labeled, with 2.8% identified as active and 91.4% as inactive. On average, 158 compounds were tested per bioassay, and approximately 55 different bioassay results were available for each compound (Fig. 1c). In determining the inclusion criteria for InertDB, we conservatively interpreted PubChem assay results: if a compound was inconsistently annotated as both active and inactive within the same bioassay, it was classified as active. Notably, literature-derived assay results, predominantly annotated as either unspecified (3.7%) or inconclusive (2%), required manual review for accurate classification [30]. During the review, compounds showing 50% of maximal activity (AC_50_) values at concentrations ≤ 1,000 µM were classified as active; otherwise, they were considered inactive (Supplementary Fig. 1).

**Fig. 2:**
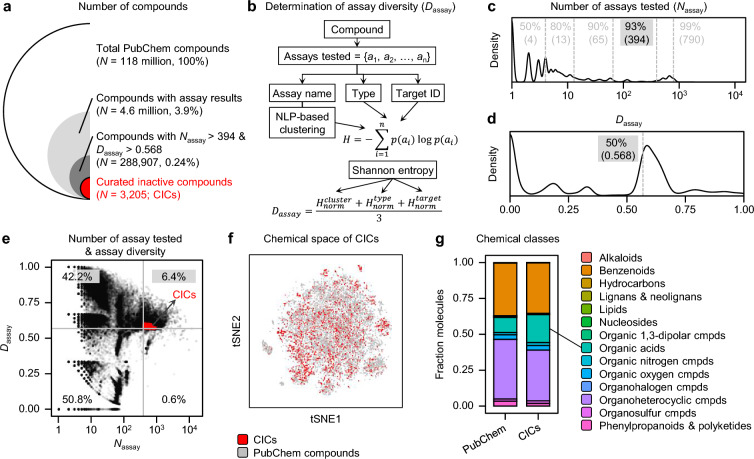
Collection of curated inactive compounds. **a**. The number of compounds in PubChem with assay results and CICs. **b**. A schematic diagram describing the process for the determination of assay diversity (*D*_assay_). **c**. Distribution of number of assays tested (*N*_assay_) across the PubChem compounds with assay results. **d**. Distribution of *D*_assay_ across the PubChem compounds with assay results. **e**. Compounds with *N*_asasy_ > 394 and *D*_assay_ > 0.568 were selected as CICs. **f**. Chemical space of CICs compared with that of PubChem compounds using t-SNE algorithm applied on chemical fingerprints. **g**. Chemical structures of compounds from PubChem or CICs were classified using ClassyFire

Importantly, we aimed to select chemicals that demonstrated ineffectiveness across a sufficiently diverse range of bioassays. To ensure the reliability of the selected inactive compounds, we developed a metric to evaluate the diversity of bioassays in which the compounds were tested, called *D*_assay_ (Fig. 2a). Relying solely on the number of bioassays (*N*_assay_) can be biased; for example, 5-methyldeoxycytidine (CID 1835) has 70 different bioassay results in PubChem, all derived from the cell growth inhibition assay performed using NCI-60 cell lines in the Human Tumor Cell Lines Screen project [31]. Such bias in a specific assay type does not adequately reflect true bioassay diversity. To address this, we employed a natural language processing (NLP)-based cluster analysis of bioassay names to determine assay diversity (*D*_assay_) (Fig. 2b). Using NLP-based embeddings, 1,621,918 bioassay names in PubChem were categorized into 8,976 distinct clusters (Supplementary Fig. 2). In addition, each bioassay was classified into 17 unique assay types and 16,669 unique target IDs according to the PubChem annotations (Supplementary Fig. 2). Based on the categorized bioassays, the *D*_assay_ of each chemical was determined by averaging normalized Shannon entropy values for the cluster (${H}_{norm}^{cluster}$), assay type (${H}_{norm}^{type}$), and assay target ID (${H}_{norm}^{target}$), thus assessing the information content as a measure of bioassay diversity [32, 33] (Fig. 2b). FDA-approved drugs, which undergo extensive biological testing, exhibited significantly higher *D*_assay_ values (*P* < 0.0001) compared to randomly sampled PubChem compounds (Supplementary Fig. 3). In contrast, compounds with low *D*_assay_ values were predominantly screened within highly redundant assay sets, such as gene expression assays in a single cell type or viability assays in cancer cell lines (Supplementary Fig. 4). These findings demonstrate the reliability of assay diversity metric in identifying compounds assessed across a wide range of biological contexts.

In InertDB, both *N*_assay_ and *D*_assay_ for each chemical were used as inclusion criteria. The *N*_assay_ distribution per compound indicated that the 50th, 80th, 90th, and 99th percentiles were 4, 13, 65, and 790, respectively (Fig. 2c). The 93rd percentile (394 assays), representing the rightmost local maxima of the distribution, was selected as the *N*_assay_ threshold for inclusion. For *D*_assay_, to include compounds tested across a diverse range of bioassays, the median value of 0.568 was chosen as the cutoff, representing the significant diversity (Fig. 2d). Consequently, 6.4% of the chemicals with assay results in the PubChem met this criterion (Fig. 2e). From this subset, 3,205 compounds were determined to be inactive in all tested bioassays, referred to as curated inactive compounds (CICs) (Fig. 2a; Supplementary Fig. 1).

Upon exploring the chemical space of CICs with that of PubChem compounds, we observed a significant overlap (Fig. 2f). Detailed analysis of chemical classes revealed that benzenoid and organic heterocyclic compounds were the most prevalent among the CICs, accounting for 35.2% and 35.3% respectively (Fig. 2g). These classes were similarly predominant in PubChem, constituting 36.7% and 41.5% of the database, respectively. However, organic acids and their derivatives were particularly overrepresented in the CICs, comprising 19.5% compared to 10.5% in PubChem (Fig. 2g). Additionally, organic nitrogen compounds, while representing a smaller proportion, exhibited a slight increase in CICs (2.1%) relative to their presence in the entire PubChem database (1.6%). This subtle yet notable difference underscores the nuanced shifts in chemical class distributions between inactive compounds and the broader chemical entries in PubChem.

### Chemical characteristics of CICs

**Fig. 3:**
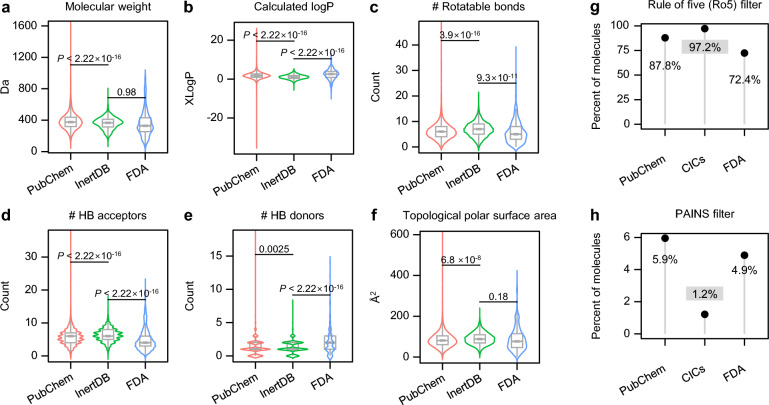
Chemical characteristics of CICs. **a-f**. Comparison of physicochemical properties of CICs with those of PubChem compounds and FDA-approved drugs. **g**, Proportion of compounds complying with the Rule of Five (Ro5) for PubChem compounds, CICs, and FDA-approved drugs. **h**, Proportion of PAINS in each subset

We next compared the molecular properties of CICs with those of PubChem compounds and FDA-approved drugs to identify the potential biases in the chemical space of inactive compounds (Fig. 3). The physicochemical properties of CICs closely matched those of FDA-approved drugs, with no significant differences in molecular weight (MW) or topological polar surface area (TPSA) (Figs. 3a and f). However, there were notable distinctions in the numbers of hydrogen bond (HB) acceptors and donors (both *P* < 2.22 × 10^–16^; Figs. 3d and e), which may influence the hydrophobicity of the compounds, as indicated by significant differences in calculated logP (XLogP) values [34] (Fig. 3b). These findings emphasize the importance of hydrogen-bonding interactions in modulating enzyme functions and receptor activations by ligand binding [35]. When applying the Rule of Five (Ro5) for evaluating drug-likeness properties [36, 37], 97.2% of the CICs met the Ro5 criteria (Fig. 3g). The Ro5 compliance among CICs was notably higher than that among randomly selected PubChem compounds (87.8%). This suggests that CICs exhibit promising drug-like characteristics, enhancing their potential application in machine learning-based predictive models.

Pan-assay interference compounds (PAINS) are chemical entities that often produce false-positive results in HTS by affecting various bioassays through nonspecific mechanisms, including redox activity, aggregation, and fluorescence interference [38]. When we calculated the proportion of PAINS in each chemical set, approximately 5.9% of the compounds in PubChem were identified as PAINS. In contrast, while only 1.2% of the 3,205 CICs fell into this category, suggesting effective filtering of PAINS during the collection of CICs. Furthermore, about 4.9% of FDA-approved drugs were PAINS, consistent with previous reports [39]. Notably, the PAINS found among FDA-approved drugs were identified in conventional low-throughput experimental settings rather than target-based HTS [39]. These insights suggest that CICs can improve the applicability of machine learning-based predictive models by minimizing risks of off-target effects and PAINS while preserving favorable physicochemical properties.

### Generative AI for Inactive compounds

**Fig. 4:**
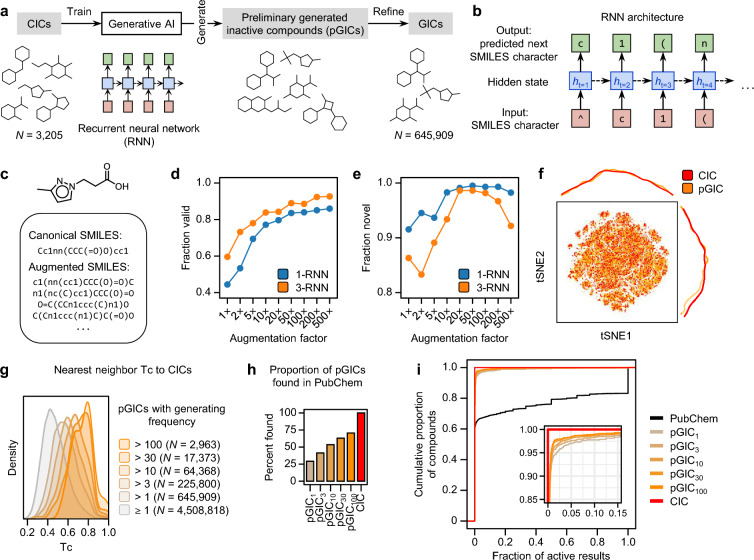
Deep generative AI model for proposing potential inactive compounds. **a**, Generative AI for producing potential inactive compounds from CICs. **b**, A schematic diagram describing input and output of RNN-based generative model. **c**. SMILES augmentation. **d**,**e**. Performances of generative AI for fraction valid (**d**) and fraction novel (**e**) among generated SMILES varying the augmentation factor and the number of RNN layers. **f**, Chemical space of CICs and pGICs. **g**, Nearest neighbor chemical similarity (Tc) to CICs by pGICs with different generating frequencies. **h**, Proportion of pGICs found in PubChem across different generating frequency subsets. **i**, Cumulative distribution of the fraction of active assay results for individual compounds in different pGIC subsets

While CICs are curated from PubChem as biologically inactive small molecules, the chemical diversity associated with them may be insufficient for broad application in AI-based predictive modeling [12]. To expand the chemical space of the 3,205 CICs, we developed a generative AI model designed and trained to propose the potential inactive compounds (Fig. 4a). Recurrent neural network (RNN)-based generative models have shown success in virtually generating chemical libraries of lead-like molecules [40] and psychoactive substance analogs [41], particularly in low-data regimes [42]. The RNN-based generative AI models predict the next SMILES character given a sequence of preceding SMILES characters (Fig. 4b). In this context, SMILES augmentation, which represents the same chemical structure using various SMILES strings, is crucial for training a robust and reliable generative AI model from a limited number of reference SMILES strings [43] (Fig. 4c).

To develop an optimal generative AI for inactive compounds, we trained and evaluated RNN architectures with one or three layers, varying SMILES augmentation factor ranging from 2- to 500-fold. Notably, as the augmentation factor increased, the proportion of syntactically valid SMILES strings improved, particularly in the three-layer networks (3-RNN) compared to the single-layer networks (1-RNN) (Fig. 4d). Syntactically valid SMILES strings can be correctly converted back into chemical structures, whereas insufficient training may produce SMILES strings with incorrect molecular valency or improper ring closures, rendering them uninterpretable as valid chemical structures [43]. Interestingly, even a tenfold augmentation exhibited a validity exceeding 80%, sufficient for generating SMILES strings (Fig. 4d; Supplementary Fig. 3).

Furthermore, the fraction of novel compounds in the chemical set generated by the 1-RNN was consistently higher across all levels of augmentation factors compared to that produced by the 3-RNN. Notably, the fraction of novel SMILES generated by the 3-RNN was comparable to that of the 1-RNN within the 20- to 100-fold augmentation range (Fig. 4e). A decline in the novelty fraction beyond 100-fold augmentation was attributed to potential model overfitting. Additional metrics, including uniqueness and scaffold similarity, revealed that uniqueness stabilized above 20-fold augmentation, while scaffold similarity declined significantly beyond 50-fold augmentation (Supplementary Fig. 5). To balance validity and novelty, we selected the 3-RNN model trained with 50-fold augmented SMILES to generate a dataset comprising 10 million SMILES strings, representing 4.5 million unique potential inactive compounds, referred to as preliminary generated inactive compounds (pGICs). A t-SNE mapping of the chemical space revealed a significant overlap between the pGICs and CICs (Fig. 4f). The physicochemical properties of pGICs showed notable alignment with those of CICs, particularly in MW and TPSA distributions (Supplementary Fig. 6).

Generative models often produce compounds that closely resemble their training datasets [42]. In some cases, certain SMILES strings corresponding to a single chemical structure were generated over 1,000 times in the 10 million iterations (Supplementary Fig. 7). To characterize and refine the pGICs and enhance their quality, we analyzed the generating frequency of SMILES strings produced by the CIC-trained generative AI. Based on their generating frequencies, pGICs were categorized into subsets as pGIC_100_, pGIC_30_, pGIC_10_, pGIC_3_, pGIC_1_, representing the pGICs generated more than 100, 30, 10, 3 times, and more than once, respectively, out of 10 million iterations. These subsets comprised 2,963, 17,373, 64,368, 225,800, and 645,909 compounds, respectively, from a total of 4.5 million unique pGICs (Fig. 4g). Higher generating frequencies were positively correlated with greater chemical similarity to nearest neighbor CICs, as measured by the Tanimoto coefficient (Tc). Furthermore, the likelihood of a compound being found in PubChem increased with generation frequency. For example, 70.6% of compounds in the pGIC_100_ subset were listed in PubChem, compared to only 29.2% of compounds in the pGIC_1_ subset (Fig. 4h). Given that substantial numbers of pGICs were listed in PubChem, we analyzed the cumulative distribution for fractions of active bioassay results on pGICs found in PubChem. High-frequency pGICs exhibited significantly lower fractions of active bioassay results compared to compounds randomly sampled from PubChem, indicating that pGICs are generally enriched for inactive compounds (Fig. 4i). Based on these results, we compiled compounds from pGIC_10_ subset to create a refined dataset of 64,368 compounds, referred to as generated inactive compounds (GICs). Together with the 3,205 CICs curated from PubChem, these generative AI-based GICs constitute InertDB, a comprehensive database designed to advance predictive modeling and virtual screening in drug discovery.

### Comparison of InertDB with dark chemical matter

To assess the uniqueness and potential complementarity of InertDB, we conducted a detailed comparison with the dark chemical matter (DCM) dataset, which consists of compounds that consistently remained inactive across 234 Novartis assays and 429 PubChem assays from the NIH Molecular Libraries Program [44]. Similar to InertDB, DCM represents compounds with inactivity across over a hundred biological assays. The DCM dataset contains 139,352 compounds, while InertDB contains 649,114 compounds, with 16,943 compounds (2.6% of InertDB compounds) shared between the two datasets (Supplementary Fig. 8a).

Our chemical space analysis revealed substantial similarities between InertDB and DCM while also highlighting notable distinctions. While the overall chemical distributions overlapped, DCM exhibited concentrated regions, suggesting that certain substructures were overrepresented (Supplementary Fig. 8b). To further investigate these differences, we examined chemical class composition, finding that InertDB contains a higher proportion of benzenoid and organic acid compounds, whereas DCM is enriched in organoheterocyclic compounds (Supplementary Fig. 8c). At the scaffold level, we identified 147,109 unique scaffolds across both datasets, with benzylaniline, diphenylthiohydroxylamine, and benzoimidazole as core structures common to both (Supplementary Fig. 8d). However, 4,800 scaffolds (3.3%) were significantly enriched in one dataset over the other (Supplementary Fig. 8e). Specifically, InertDB is enriched in dioxaspiroundecane and dioxinylpyrrolidine scaffolds, which are largely absent from DCM (Supplementary Fig. 8f). Conversely, DCM contains a higher proportion of phenylthiazole, phenylimidazole, benzylazetidine, and benzylthiomorpholine scaffolds, which are underrepresented in InertDB (Supplementary Fig. 8 g). Despite these structural differences, both datasets exhibit comparable PAINS-filter compliance (1.2% in DCM) and Ro5 adherence (97.1% in DCM), reinforcing their suitability as starting points for virtual screening. These findings indicate that while InertDB and DCM share a subset of compounds, their distinct chemical compositions make them highly complementary resources.

### Validation Study of InertDB

Next, we performed a validation study to investigate the efficacy of InertDB in enhancing the performance of machine learning models for predicting the biological activity of chemical compounds. We used well-established benchmark datasets, LIT-PCBA [45] and Maximum Unbiased Validation (MUV) [46]. The LIT-PCBA dataset provides activity annotations for 15 bioassays, while the MUV dataset includes annotations for 17 bioassays. These bioassays encompass a broad range of targets, including G-protein-coupled receptors, nuclear receptors, and kinases, ensuring diverse assay coverage (Supplementary Table 1).

**Fig. 5:**
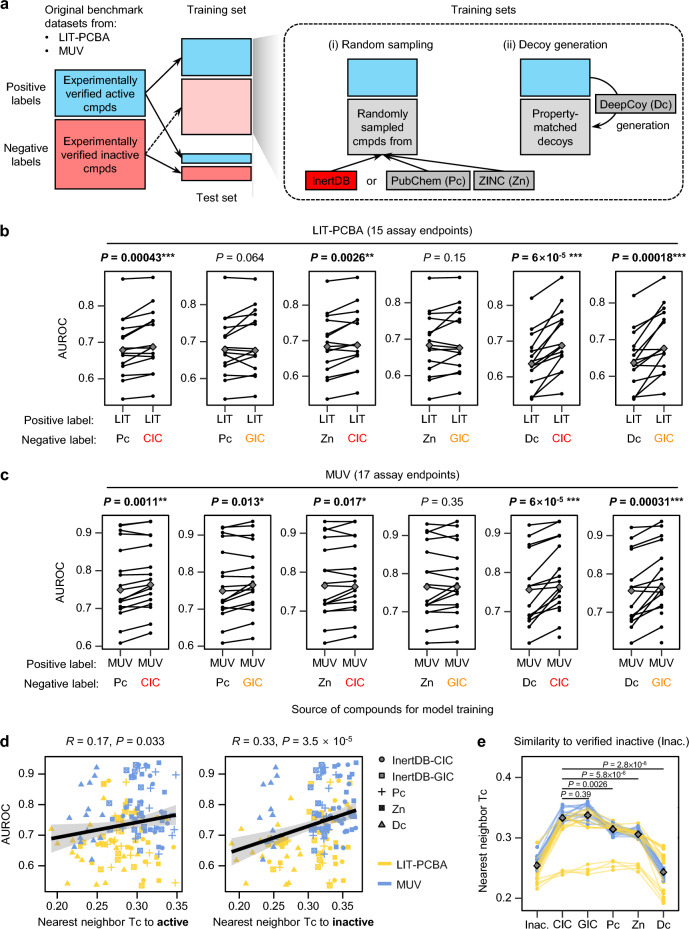
Validation study of InertDB. **A**. Schematic diagram describing strategies for preparing training dataset to compare efficacy of random sampling and decoy generation methods. **b**,**c**. Mean predictive performances for LIT-PCBA (**b**) and MUV (**c**) datasets. Each model was constructed by training the random forest-based classifier with ECFP4, with different datasets as sources for positive and negative labels. Performance was evaluated on the hold-out test set consisting of original verified active and inactive compounds from each benchmark dataset. The performances are compared in area under the receiver operating characteristic curve (AUROC) values. A higher AUROC value reflects superior classification performance, indicating that the predictive model can more effectively distinguish between active and inactive compounds. Each data point represents the mean AUROC value from 100 random splits for an individual assay endpoint in the benchmark dataset. Gray squares indicate median values. Statistical significance between paired assay endpoints (connected by lines) was determined using a paired Wilcoxon test: *P < 0.05, **P < 0.01, and ***P < 0.001. **d** Spearman correlation between model performance and chemical similarity (nearest neighbor Tc) of negative-label compounds in the training set to verified active (left) or inactive (right) compounds from the original benchmark datasets. **e** Mean chemical similarity (nearest neighbor Tc) between verified inactive compounds (Inac.) and compounds from InertDB (CIC and GIC subsets), PubChem (Pc), ZINC (Zn), and DeepCoy-generated decoys (Dc) for each assay endpoint

To evaluate the efficacy of InertDB, we implemented two different modeling strategies: (i) training models with active compounds verified in LIT-PCBA or MUV, and inactive compounds randomly selected from either the CIC or GIC subsets of the InertDB, PubChem, or ZINC, and (ii) training models with verified active compounds, and inactive compounds randomly selected from AI-generated property-matched decoys [23] (Fig. 5a). Each model was validated using an identical hold-out test set composed of verified active and inactive compounds derived from LIT-PCBA or MUV datasets to ensure the robustness and comparability of the results.

When the random forest-based classifier with ECFP4 was applied to validation analyses, models incorporating compounds randomly sampled from InertDB, particularly the CIC subset, demonstrated significantly improved performance, as measured by area under the receiver operating characteristic curve (AUROC), compared to those using compounds from PubChem (*P* = 0.00043 for LIT-PCBA and *P* = 0.0011 for MUV) or ZINC (*P* = 0.0026 for LIT-PCBA and *P* = 0.017 for MUV) (Figs. 5a and b). Additionally, models trained with the GIC subset of InertDB showed a significant improvement in performance specifically within the MUV dataset compared to those trained with PubChem (*P* = 0.013; Fig. 5c). Similar trends were observed when evaluating model performance using two additional metrics, Matthews correlation coefficient (MCC) and balanced accuracy (Supplementary Figs. 9 and 10). Across both LIT-PCBA and MUV benchmarks, models trained with the CIC subset of InertDB consistently outperformed those trained with compounds randomly sampled from PubChem. This improvement in model performance underscores the potential of InertDB for predictive modeling in low-data settings.

To further evaluate the efficacy of model training using inactive compounds randomly selected from InertDB, we conducted a comparative analysis against decoy compounds generated by the DeepCoy model [23]. The DeepCoy model, trained on the ZINC database, generates property-matched decoys derived from the structures of active compounds, serving as potential inactive compounds (Fig. 5a). Notably, our analysis revealed that models trained with either the CIC or GIC subsets of InertDB consistently outperformed those trained with DeepCoy-generated decoys within LIT-PCBA (*P* = 6 × 10^–5^ for CIC and *P* = 0.00018 for GIC) and MUV (*P* = 6 × 10^–5^ for CIC and *P* = 0.00031 for GIC) datasets (Figs. 5b and c, Supplementary Figs. 9 and 10). The DeepCoy model was initially developed to address potential biases inherent in traditional decoy datasets like DUDE-E. Originally designed for structure-based virtual screening against specific targets, DeepCoy has increasingly been applied in ligand-based virtual screening as well [25–28]. Although DeepCoy offers a realistic framework for evaluating novel structure-based virtual screening approaches by generating decoy compounds that closely mimic the physicochemical properties of active compounds, the decoys generated by DeepCoy might not adequately represent the diversity of inactive compounds in a ligand-based setting, thus may not be the optimal choice for creating ligand-based predictive models [23, 24].

To ensure a rigorous evaluation of InertDB’s effectiveness, we further compared models trained on verified active and inactive compounds from the original benchmark datasets (LIT-PCBA and MUV) as baseline (BL) models. Using identical train-test splits, we assessed whether replacing verified inactive compounds with InertDB subsets (CIC or GIC) influenced model performance. In the LIT-PCBA benchmark, no significant difference was observed between BL and InertDB-based models (*P* = 0.52 for CIC and *P* = 0.85 for GIC), while in the MUV benchmark, replacing inactive compounds with CIC led to a slight but significant improvement (*P* = 0.0032) (Supplementary Fig. 11), likely due to the smaller training set size in MUV. Given that these models were evaluated under identical conditions, the results further support the utility of InertDB as a reliable resource for augmenting or replacing inactive compounds in predictive modeling.

To further investigate the factors contributing to improved model performance, we analyzed the structural similarity between compounds sampled from each source and verified active or inactive compounds for each assay endpoint in the LIT-PCBA (Supplementary Fig. 12) and MUV (Supplementary Fig. 13) benchmarks. Model performance correlated more strongly with similarity to verified inactive compounds (*R* = 0.33, *P* = 3.5 × 10^–5^) than that to verified active compounds (*R* = 0.17), highlighting the importance of selecting inactive compounds structurally aligned with verified inactive compounds when constructing training datasets (Fig. 5d). Notably, while CIC and GIC compounds showed no significant structural differences (*P* = 0.39), CIC compounds exhibited significantly higher similarity to verified inactive compounds than compounds sampled from PubChem (*P* = 0.0026) and ZINC (*P* = 5.8 × 10⁻⁶), or decoys generated using the DeepCoy model (*P* = 2.8 × 10⁻⁸). These findings suggest that the structural alignment between InertDB compounds and experimentally verified inactive compounds contributes to the enhanced predictive performance observed in InertDB-trained models. Collectively, our results suggest that InertDB, with its refined selection of inactive compounds from PubChem, serves as an effective alternative for developing predictive models by providing reliable inactive compounds.

## Discussion

InertDB is a valuable resource for AI-assisted drug discovery, serving as an extensive virtual screening library. An effective virtual screening library should possess a diverse array of chemical structures to enhance coverage and improve the probability of identifying pharmacologically active compounds [47]. It is crucial for the chemicals within these libraries to exhibit drug-like characteristics, including adherence to the Ro5, to ensure they possess favorable pharmacokinetic profiles suitable for therapeutic development [48]. The majority of compounds within InertDB adhere to the Ro5 criteria, indicating they exhibit the physicochemical characteristics desirable for orally administered drugs. Previous studies have indicated that incorporating a collection of compounds with no known biological activity into a virtual screening library can reduce the risk of undesired off-target effects in drug discovery [44, 49]. InertDB is particularly advantageous for this purpose, as its compounds have been evaluated across diverse bioassays and consistently classified as inactive in PubChem. Additionally, InertDB exhibits a lower proportion of PAINS compared to chemical databases such as PubChem and ZINC, suggesting a reduced likelihood of selecting false positives during virtual screening. A comparative analysis with the DCM dataset [44] further highlights InertDB’s complementary nature. While both datasets capture consistently inactive compounds, they exhibit distinct chemical compositions, with InertDB enriched in benzenoid and organic acid compounds and DCM containing more organoheterocyclic scaffolds. Despite these differences, both datasets share similar Ro5 adherence and PAINS-filter compliance, reinforcing their suitability for virtual screening. Leveraging both datasets could provide a broader and more diverse chemical landscape, improving predictive modeling and drug discovery efforts. Thus, InertDB offers beneficial characteristics for virtual screening, including structural diversity, favorable drug-like properties, minimized off-target activities, and a lower risk of false positives.

InertDB is also applied to the development of diverse predictive machine learning models by providing data on inactive compounds. In validation studies using random forest classifiers with the LIT-PCBA and MUV datasets, InertDB demonstrated improved performance in selecting inactive compound sets compared to property-matched decoy generation or random sampling from PubChem or ZINC. This improvement was particularly attributed to the higher structural similarity of InertDB compounds to experimentally verified inactive compounds, which strongly correlated with enhanced predictive accuracy. The publication bias favoring biologically active compounds has resulted in a deficiency of biological activity data for inactive compounds, or negative data, posing a significant challenge in constructing accurate predictive models [10, 12]. InertDB effectively addresses this gap, playing a role as a valuable resource for inactive compounds and facilitating the development of more robust machine learning-based predictive models. To enhance accessibility and usability, InertDB is freely available via the repository (https://github.com/ann081993/InertDB), allowing researchers to access the curated and generated datasets directly. Additionally, scripts for generating additional GICs using a pre-trained deep generative AI model are provided, enabling users to further expand the chemical space based on their specific research needs. This open-access approach ensures that InertDB can be easily integrated into workflows for virtual screening, predictive modeling, and other AI-based drug discovery applications.

## Conclusions

Taken together, InertDB represents a significant advancement in chemical databases by addressing the critical need for negative data. By rigorously identifying 3,205 CICs from PubChem and expanding its chemical space with 64,368 GICs using deep generative AI, InertDB improves the accuracy of AI-based predictive models. This database mitigates the publication bias toward active compounds and reduces false positives in virtual screening, thereby improving the robustness of predictive modeling and the reliability of biological activity predictions. InertDB will be a critical resource for the development of more accurate and reliable machine learning models.

## Methods

### PubChem database

To collect inactive compounds, the complete bioassay data was downloaded from PubChem database via FTP site [8, 29] (https://ftp.ncbi.nlm.nih.gov/pubchem/Bioassay/). As of March 2024, PubChem contained 1,621,918 distinct bioassays, involving 4,627,360 compounds each with at least one assay result, and a total of 263,051,872 bioassay results.

### Cluster analysis on assay name embeddings

Natural language processing (NLP) techniques and the subsequent cluster analysis were employed to categorize the bioassays based on their names [50]. Bioassay names were retrieved via PubChem FTP and were then encoded into numeric vectors using TinyBioBERT [51], a pretrained language model for biomedical context. TinyBioBERT, a distilled version of BioBERT [52] v1.1, with four hidden layers of 312 unit each has been trained on over one million PubMed articles. As a result, each bioassay name was represented as 312-dimensional numeric embeddings. The model was available through python library *transformers* (https://huggingface.co/nlpie/tiny-biobert).

Next, the HDBSCAN algorithm was applied on the assay name embeddings with min_cluster_size = 20 and cluster_selection_epsilon = 0.03, which resulting in the categorization of bioassays into distinct clusters based on their names [53]. For two-dimensional (2D) visual representation, the assay name embeddings were further processed using the Uniform Manifold Approximation and Projection (UMAP) algorithm [54], allowing for the visualization of complex data in a simplified 2D space.

### Assay diversity

To quantitatively assess the diversity of assays in which a given compound was tested, we defined a metric referred to as an ‘assay diversity’ (*D*_assay_). *D*_assay_ is quantified as the arithmetic mean of normalized Shannon entropy values for three distinct aspects of bioassays: (1) clusters derived from NLP-based embeddings of assay names, (2) assay types, and (3) target IDs. The assay type and target ID were obtained from the bioassay annotations in PubChem. To quantify the diversity with the normalized Shannon entropy (*H*_*norm*_), the set of unique categories for PubChem bioassays associated with a given compound, $S=\{{a}_{1},{a}_{2}, \cdots , {a}_{n}\}$, was constructed, and the frequency *f*(*a*_*i*_) was defined as the frequency of the assay category *a*_*i*_ in the list. Then, the probability *p*(*a*_*i*_) was determined by scaling the frequency to the total number of bioassays (*N*) in which the compound has been tested:

1 $$p\left({a}_{i}\right)=\frac{f\left({a}_{i}\right)}{N}$$

Thus, in the context of *D*_assay_, probability *p*(*a*_*i*_) represents the proportion at which a particular category of assay was observed for given compound. Using these probabilities, Shannon entropy *H* was calculated as follows:

2 $$H=-\sum_{i=1}^{n}p\left({a}_{i}\right)\text{log}p\left({a}_{i}\right)$$

To derive the normalized Shannon entropy (*H*_*norm*_), the *H* value was divided by the logarithm of the number of unique categories *n*, which represents the maximum possible entropy where bioassay results were available for all categories:

3 $${H}_{norm}=\frac{H}{{\text{log}}_{2}n}$$

The *H*_*norm*_ value ranges from 0 to 1, where 0 indicates no diversity meaning that all assays performed against a given compound fell into a single category, whereas 1 indicates maximum diversity, where data were equally available to all assay categories in PubChem bioassays. Collectively, *H*_norm_ was calculated independently after given assays were categorized by the cluster (8,976 unique clusters), the assay type (17 unique types), and the target ID (16,669 unique IDs). Finally, the assay diversity, *D*_assay_, was determined by averaging those three normalized entropies:

4 $${D}_{assay}=\frac{{H}_{norm}^{cluster}+{H}_{norm}^{type}+{H}_{norm}^{target}}{3}$$

### Inclusion criteria for the determination of curated inactive compounds (CICs)

We assessed the number of bioassays (*N*_assay_) tested for each compound in PubChem, as well as the assay diversity *D*_assay_. By analyzing the multimodal distribution of *N*_assay_ and *D*_assay_ across all compounds in PubChem, we determined the threshold that encompassed the largest local maxima. In PubChem, bioassay results for compounds are annotated as active, inactive, unspecified, and inconclusive [30]. The bioassay information was directly adopted when compounds were labeled as active or inactive in PubChem annotations. For compounds labeled as unspecified or inconclusive, despite having available bioassay results linked to PubMed references, we manually curated their activity outcomes from the literature. Accordingly, compounds exhibiting 50% of the maximal activity (AC_50_) at concentrations less than or equal to 1,000 µM were labeled as active; otherwise, they were labeled as inactive. By applying the above criteria, we identified 3,205 compounds that were consistently recorded as inactive across all bioassay results. These compounds are termed curated inactive compounds (CICs).

### Comparison of chemical datasets

We compared characteristics of our curated CICs with those of the open chemical databases, PubChem [8] and ZINC20 [13]. The list of FDA-approved drugs was obtained from ZINC20 (https://zinc20.docking.org/). Using the python library RDKit, we calculated the physicochemical properties and determined the proportion of compounds flagged by the Pan-Assay Interference Compounds (PAINS) filter. To visualize the chemical space, compounds were represented as 1024-dimensional vectors using the Extended Connectivity Fingerprint (ECFP) with a radius of 4 (ECFP4) [55]. These vectors were then reduced to 2D using the t-Distributed Stochastic Neighbor Embedding (t-SNE) algorithm.

Chemical similarity was quantified using the Tanimoto coefficient (Tc), which is calculated by dividing the number of shared features by the sum of the unique features in ECFP4 of both compounds. This coefficient provides a measure of similarity between two chemical structures, ranging from 0 (no similarity) to 1 (identical or complete similarity). For chemical classification, we employed ClassyFire [56] (http://classyfire.wishartlab.com/), an automated tool that classifies compounds based on standardized chemical ontology. To facilitate a comparative analysis, a randomly sampled subset of 50,000 compounds from PubChem and ZINC20 was used for calculating physicochemical properties, visualizing chemical space, and performing chemical classification. This methodology allowed us to efficiently compare large datasets while maintaining computational feasibility.

### Generated inactive compounds (GICs)

To expand the chemical space of inactive compounds, we trained a recurrent neural network (RNN)-based generative AI model with a dataset of 3,205 CICs as a reference set, enabling the generation of potential inactive compounds. We constructed a character-level generative AI using long-short term memory (LSTM) layers, with SMILES (Simplified Molecular Input Line Entry System) notation as both the input and output [40]. We constructed the generative AI with either one or three LSTM layers to determine the optimal architecture.

To enhance model performance, especially when trained on a small number of compounds, we adopted SMILES augmentation, varying the degree of augmentation factor from twofold to 500-fold [43]. The model was implemented using python *tensorflow* framework and was trained using the Adam optimizer for up to 300 epochs, with β_1_ = 0.9, β_2_ = 0.999, and the learning rate of 0.001. To prevent overfitting, we applied early stopping with a delta of 0.001 and a patience setting of 10 epochs.

To evaluate the performance of the generative model, we calculated six metrics from the subset of 10,000 generated SMILES strings: validity, uniqueness, novelty, scaffold similarity, and fragment similarity, as previously described [57]. Validity is the metric to determine the proportion of syntactically valid SMILES strings generated by the model. A SMILES string is considered valid if it can be correctly parsed by *MolFromSmiles* function of RDKit. Uniqueness measures the proportion of unique SMILES strings among the strings generated by the model. High uniqueness indicates that the model can generate a wide variety of chemical structures without repeatedly producing the same molecule. Novelty metric is the proportion of novel SMILES strings generated by the model that are not present in the reference set. High novelty indicates that the model can produce new chemical entities that could potentially offer unexplored chemical space. Scaffold similarity is the metric for the similarity between the scaffolds of SMILES strings generated by the model and those of the reference set. The list of available scaffolds in given chemical set is obtained using *GetScaffoldForMol* function of RDKit. Then, the cosine similarity was calculated between normalized frequency of scaffolds for reference SMILES strings and generated ones. Finally, fragment similarity is the metric associated with the similarity between the substructures (fragments) of SMILES strings generated by the model and those of the reference set. The list of available fragments in given chemical set is obtained using *FragmentOnBRICSBonds* function of RDKit, then cosine similarity was calculated.

By evaluating the performance of generative AI models, we identified the 3-RNN model trained with 50-fold augmented SMILES as optimal. Using the trained generative AI model, we generated 10 million SMILES strings, from which we filtered out low-quality strings, including invalid ones and those representing inorganic compounds (e.g., azides and halides), as well as those shorter than 15 characters. Compounds present in the reference set were also excluded. This process yielded a total of 7,815,176 SMILES strings, corresponding to high-quality 4,508,818 unique chemical structures. After refining these compounds based on the generating frequency (how repeatedly the generating AI produced the SMILES string), we defined 64,368 generated inactive compounds (GICs).

Together, CICs and GICs form InertDB, a comprehensive database of inactive compounds. Additionally, to contextualize InertDB within existing chemical resources, we compared it to dark chemical matter (DCM) [44]. Chemical space and class composition analyses were performed as described above in section *Comparison of Chemical Datasets*. To compare scaffold distributions, we extracted Murcko scaffolds using RDKit and performed a chi-squared test to identify scaffolds that were significantly enriched in either dataset.

### Predictive model

To benchmark the performance of predictive models by using the CIC and GIC sets of InertDB, two datasets were utilized: LIT-PCBA [45] and Maximum Unbiased Validation (MUV) [46]. Both LIT-PCBA and MUV are curated from PubChem bioassays to create unbiased datasets to benchmark predictive models for biological activity.

For model construction, chemical structure on active and inactive compounds was encoded using ECFP4 [55]. A binary classification model was then trained based on the active/inactive labels. Model training involved three approaches for preparing training dataset: (1) extracting both active and inactive compound information from benchmark datasets (LIT-PCBA or MUV), which was also used to train baseline (BL) models, (2) using active compounds from the benchmark datasets while randomly sampling inactive compounds from other sources (InertDB, PubChem, or ZINC), or (3) using active compounds from the benchmark datasets while property-matched decoys were generated using a pretrained deep learning model as inactive compounds [23].

To compare the performances of each approach, fingerprint-based random forest models were trained with each training set and evaluated using the 20% hold-out test set for 100 repeated times. The same hold-out test set obtained from benchmark datasets was used for comparison. Model performance was primarily assessed using the area under the receiver operating characteristic curve (AUROC). Additionally, Matthews correlation coefficient (MCC) and balanced accuracy (BA) were incorporated as supplementary metrics to provide a more comprehensive evaluation of classification performance. The MCC is defined as:

5 $$MCC= \frac{TP\times TN-FP\times FN}{\sqrt{(TP+FP)(TP+FN)(TN+FP)(TN+FN)}}$$

where TP (true positive), TN (true negative), FP (false positive), and FN (false negative) represent classification outcomes. MCC ranges from -1 (total disagreement) to + 1 (perfect prediction), with 0 indicating no better performance than random chance. The BA is given by:

6 $$BA=\frac{TPR+TNR}{2}$$

where TPR (true positive rate) = TP / (TP + FN) and TNR (true negative rate) = TN / (TN + FP). To prevent model overfitting due to class imbalance, the number of negative labels in the training and test sets was limited to at most twice the number of positive labels through random undersampling.

To investigate the observed differences in model performance across datasets, we performed a chemical similarity analysis. Specifically, for each assay endpoint in LIT-PCBA and MUV, we quantified the structural similarity (nearest neighbor Tc) of compounds in InertDB (CIC or GIC subsets), PubChem, ZINC, and DeepCoy-derived decoys to those labeled as active or inactive in original benchmark dataset (verified active and inactive compounds).

## Supplementary Information

Additional file 1

## Data Availability

InertDB is publicly accessible and can be downloaded from our GitHub repository (https://github.com/ann081993/InertDB). We also provide overview and key applications of InertDB, and the scripts for generating potential inactive compounds via our repository.

## Abbreviations

AC_50_: Half-maximal Activity Concentration
AI: Artificial Intelligence
CICs: Curated Inactive Compounds
DUD-E: Database of Useful Decoys, Enhanced
GICs: Generated Inactive Compounds
HTS: High-Throughput Screening
NLP: Natural Language Processing
PAINS: Pan-assay Interference Compounds
Ro5: Rule of Five
RNN: Recurrent Neural Network
SMILES: Simplified Molecular Input Line Entry System
