# Supplementary material for: InertDB as a generative AI-expanded resource of biologically inactive small molecules from PubChem
Source: J Cheminform. 2025 Apr 10;17:49. doi: 10.1186/s13321-025-00999-1 (PMC11983867; doi:10.1186/s13321-025-00999-1)
Supplement: Supplementary file 1 — Additional file 1 [file 13321_2025_999_MOESM1_ESM.pdf]

Supplementary Information for

InertDB as a generative AI-expanded resource of biologically inactive small molecules from PubChem

Seungchan An, Yeonjin Lee, Junpyo Gong, Seokyoung Hwang, In Guk Park, Jayhyun Cho, Min Ju Lee, Minkyu Kim, Yun Pyo Kang, Minsoo Noh*

Natural Products Research Institute, College of Pharmacy, Seoul National University, Seoul, 08826, Republic of Korea

*Corresponding Author: Minsoo Noh (Email: minsoonoh@snu.ac.kr)

Keywords: Inactive compounds; Virtual screening; Synthetic negative data; Large-scale bioassay; Generative model; Predictive pharmacology

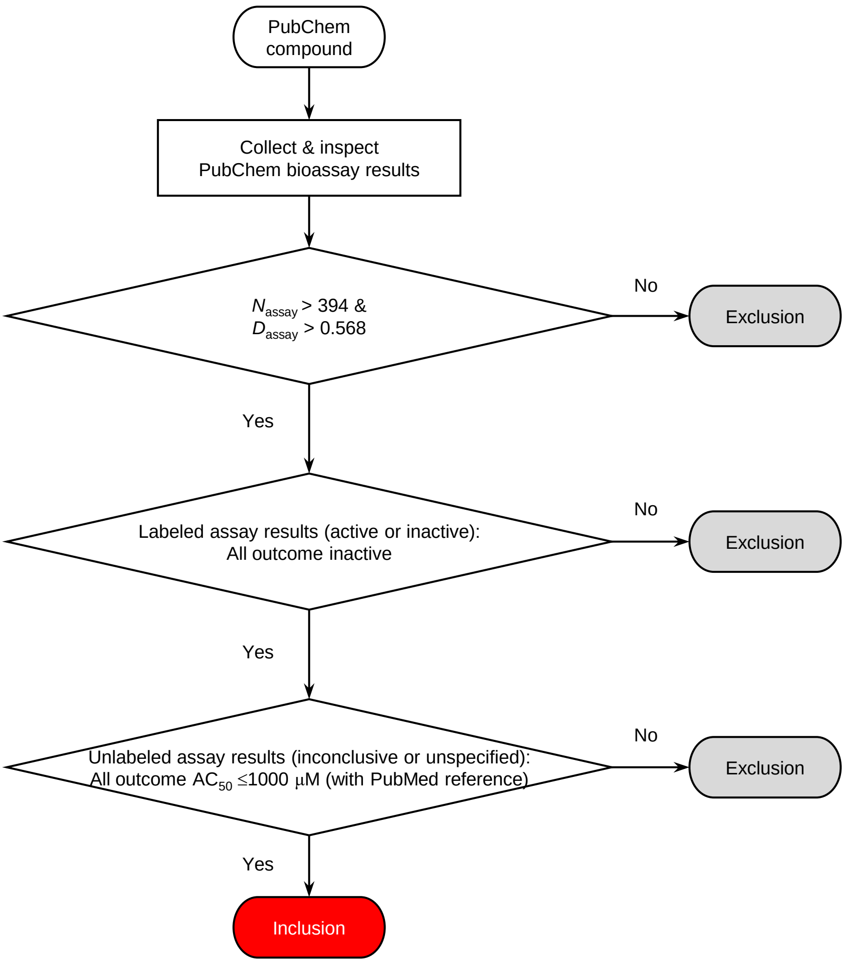

Supplementary Figure 1 | Flowchart illustrating the collection process of CICs. Starting with 4.6 million compounds from PubChem, we extracted compound information along with their assay results. Compounds that met the criteria for both N_{assay} (sufficient number of assays) and D_{assay} (diverse range of assays) were selected. From this subset, compounds with all labeled assay results classified as inactive were further refined. For these compounds, unlabeled assay results were also reviewed, and compounds were retained as CICs only if all results, including those with AC_{50} values ≤ 1,000 μM reclassified as active, were consistently inactive.

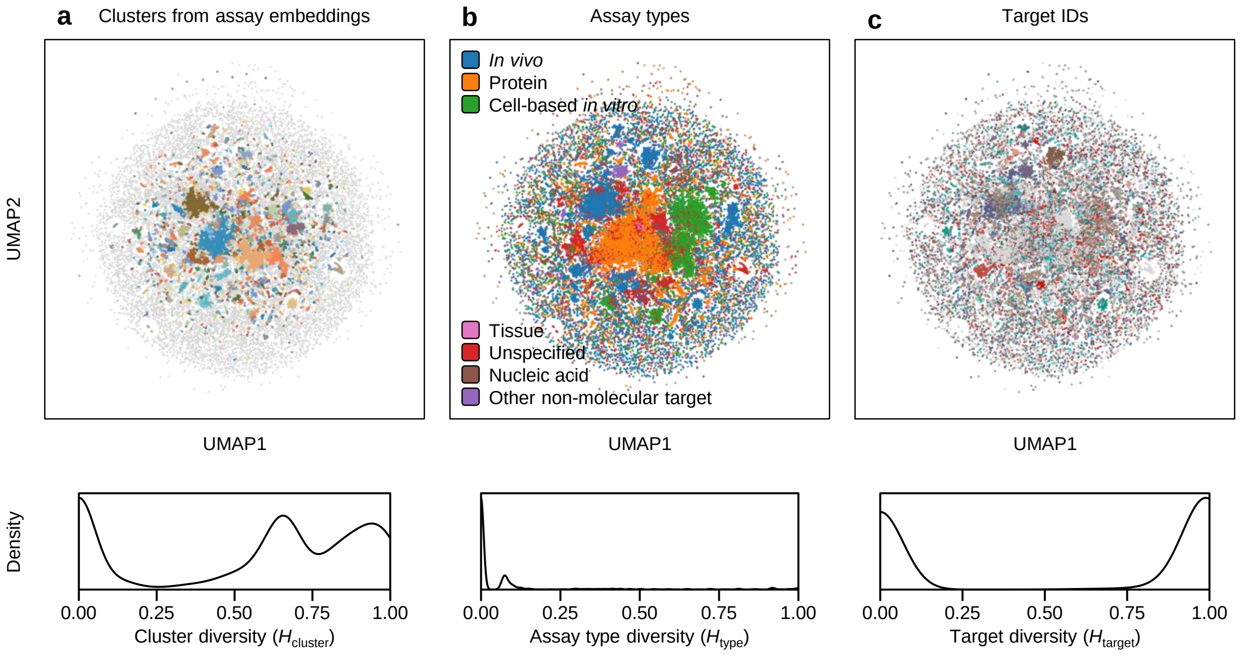

Supplementary Figure 2 | Clustering of PubChem assays based on NLP embeddings. a, Distribution of PubChem assays visualized using UMAP embeddings, colored by clusters derived from assay embeddings. **b**, UMAP visualization of PubChem assays, colored by assay types. **c**, UMAP visualization of PubChem assays, colored by target IDs. Lower panels depict the distribution of Shannon entropy-based diversity for each term (clusters, assay types, and target IDs) across PubChem compounds.

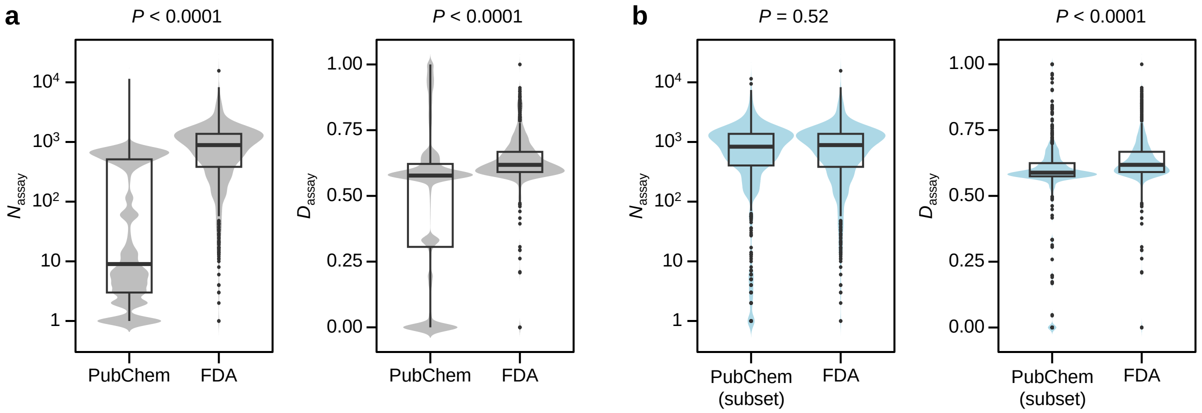

Supplementary Figure 3 | Comparison of N_{assay} and D_{assay} values between FDA-approved drugs and PubChem compounds. a, Distribution of N_{assay} and D_{assay} values for FDA-approved drugs and randomly sampled 100,000 PubChem compounds. **b**, Comparison after controlling for N_{assay} differences by extracting an N_{assay}-matched PubChem subset. Statistical significance was determined using the Wilcoxon test.

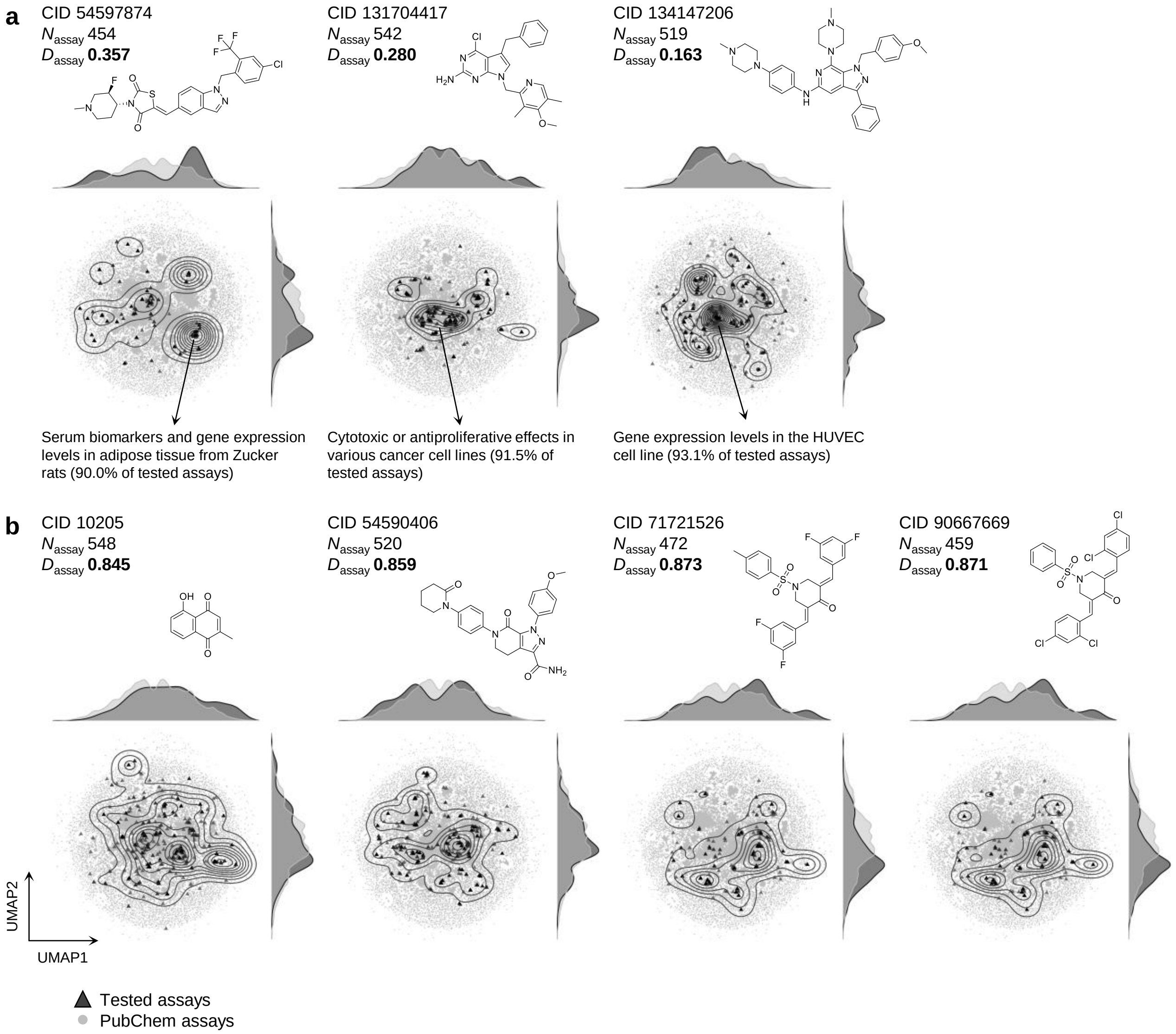

Supplementary Figure 4 | Examples of assay distribution. UMAP embeddings of PubChem assays (gray) with the tested assays for a given compound highlighted (dark gray triangles). Contour lines indicate distribution density on embeddings. **a**, Examples with low D_{assay} values. **b**, Examples with high D_{assay} values.

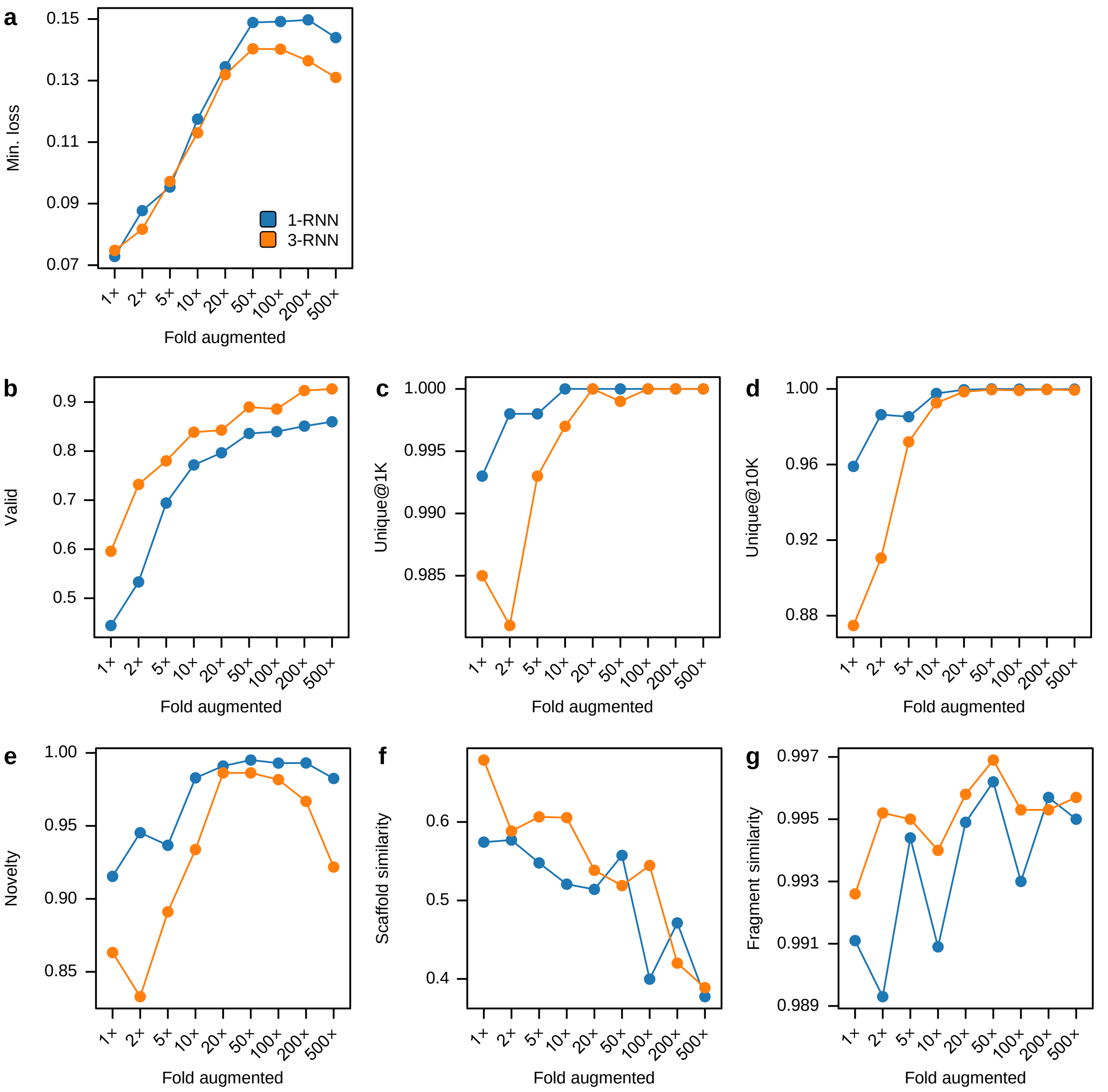

Supplementary Figure 5 | Evaluation of generative AI for producing potential inactive compounds. Generative AI models, including 1-layer RNN (1-RNN) and 3-layer RNN (3-RNN), were trained and evaluated across fold augmentations ranging from 1- to 500-fold. **a**, The results include the minimum loss achieved during training (up to 300 epochs). **b**–**g**, Six evaluation metrics for generative AI: validity of generated molecules (**b**), fraction of unique molecules among the first 1,000 generated (Unique@1K) (**c**) and the first 10,000 generated (Unique@10K) (**d**), novelty of generated molecules (**e**), scaffold similarity to the reference SMILES strings (**f**), and fragment similarity to the reference (**g**).

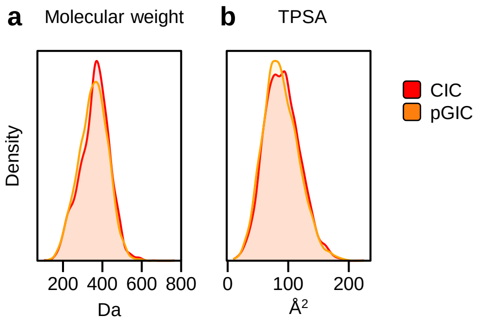

Supplementary Figure 6 | Comparison of physicochemical property distributions for generated inactive compounds. The distributions of molecular weight (**a**) and topological polar surface area (TPSA) (**b**) were compared between CICs and pGICs.

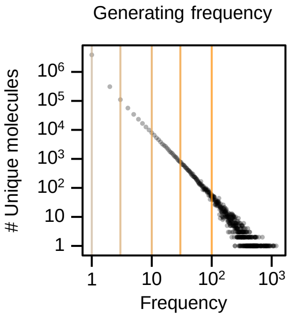

Supplementary Figure 7 | Relationship between generating frequency and the number of unique molecules per SMILES for pGICs. This figure illustrates the correlation between the generating frequency and the number of unique molecules represented by each SMILES string in the pGIC dataset.

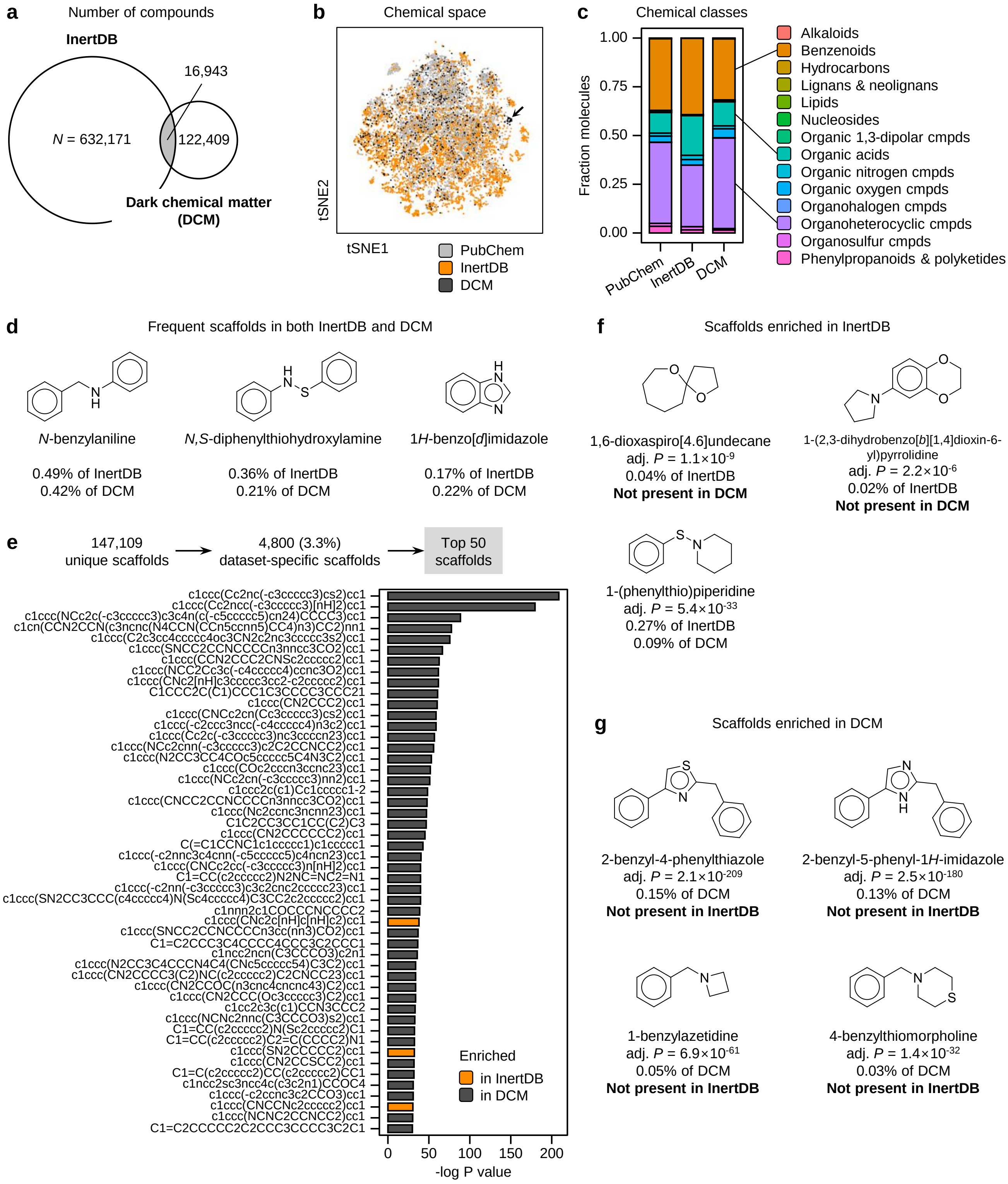

Supplementary Figure 8 | Chemical contents of InertDB and DCM. a, Venn diagram showing the number of compounds in InertDB and DCM, along with their overlapping compounds. **b**, Chemical space comparison of InertDB, DCM, and PubChem using t-SNE applied to chemical fingerprints. A subset of 10,000 compounds from each dataset was sampled for visualization. **c**, Chemical class composition of InertDB, DCM, and PubChem, determined using ClassyFire. **d**, Identification of the most common Murcko scaffolds in InertDB and DCM. **e**, Top 50 dataset-specific scaffolds identified through enrichment analysis using a chi-squared test. Representative examples of enriched scaffolds in InertDB (**f**) and DCM (**g**).

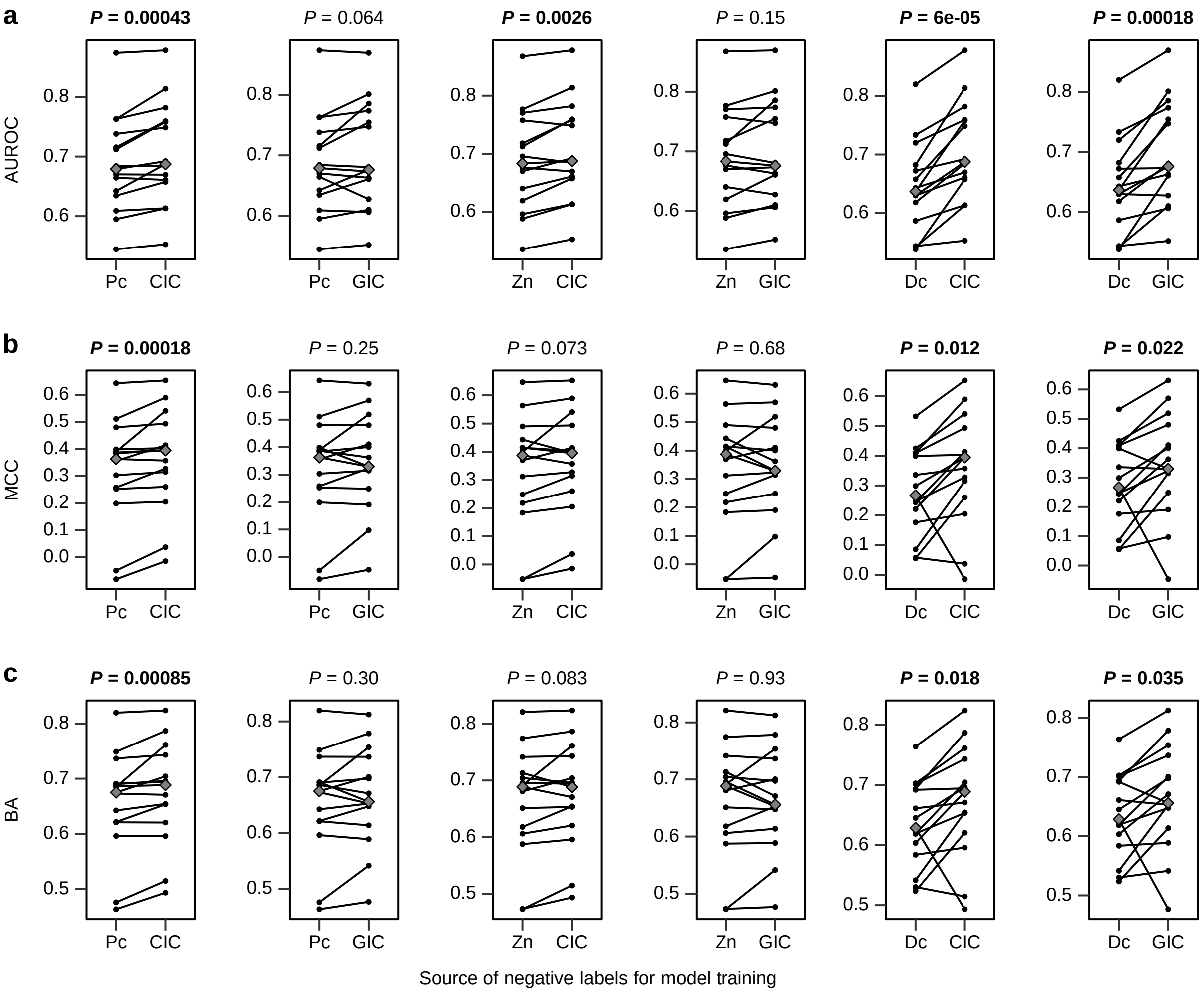

Supplementary Figure 9 | Validation study of InertDB (LIT-PCBA). Each model was trained using a random forest classifier with ECFP4 fingerprints, and performance was evaluated using AUROC (**a**), MCC (**b**), and BA (**c**). Higher values indicate superior classification efficiency. Each data point represents the mean performance across 100 random splits for an individual assay endpoint in the benchmark dataset. Gray squares denote median values. Statistical significance between paired assay endpoints (connected by lines) was determined using a paired Wilcoxon test, with significant results shown in bold. Pc: PubChem, Zn: ZINC, Dc: DeepCoy.

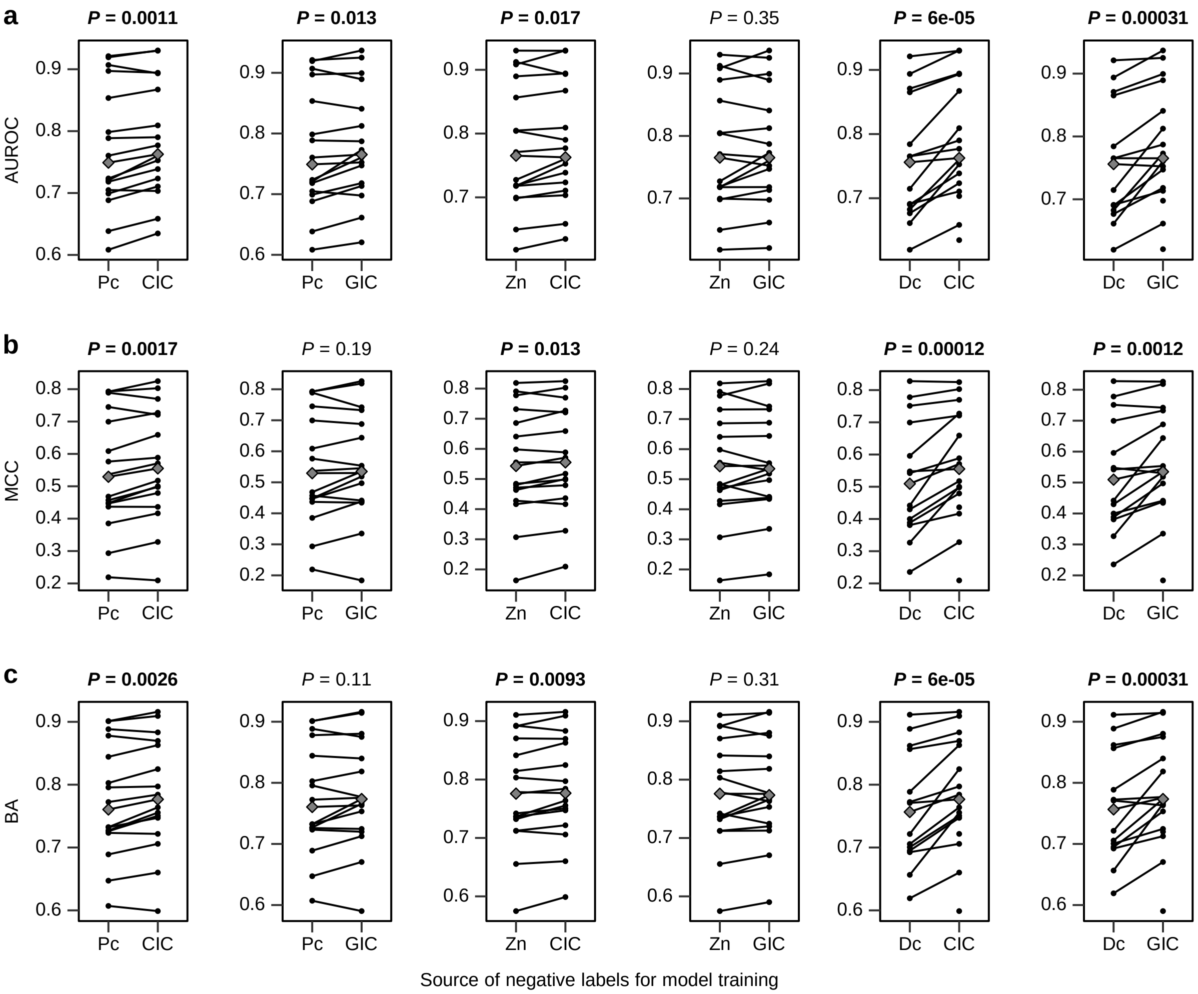

Supplementary Figure 10 | Validation study of InertDB (MUV). Each model was trained using a random forest classifier with ECFP4 fingerprints, and performance was evaluated using AUROC (**a**), MCC (**b**), and BA (**c**). Higher values indicate superior classification efficiency. Each data point represents the mean performance across 100 random splits for an individual assay endpoint in the benchmark dataset. Gray squares denote median values. Statistical significance between paired assay endpoints (connected by lines) was determined using a paired Wilcoxon test, with significant results shown in bold. Pc: PubChem, Zn: ZINC, Dc: DeepCoy.

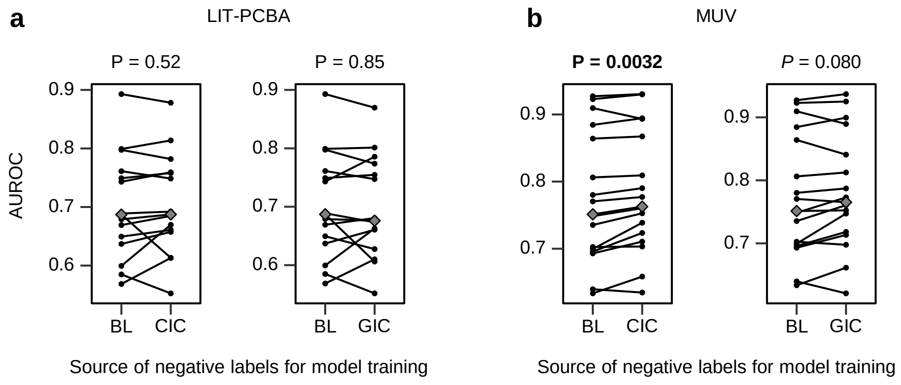

Supplementary Figure 11 | Validation of InertDB-based model compared to baseline model. Each model was trained using a random forest classifier with ECFP4 fingerprints, and performance was evaluated using AUROC. The baseline (BL) model was trained using the original training set from LIT-PCBA or MUV and evaluated on the hold-out test set. The CIC and GIC models were trained similarly, except that the negative-label compounds were replaced with the CIC or GIC subsets of InertDB, respectively. These models were then evaluated using the same hold-out test set. Gray squares denote median values. Statistical significance between paired assay endpoints (connected by lines) was determined using a paired Wilcoxon test, with significant results shown in bold.

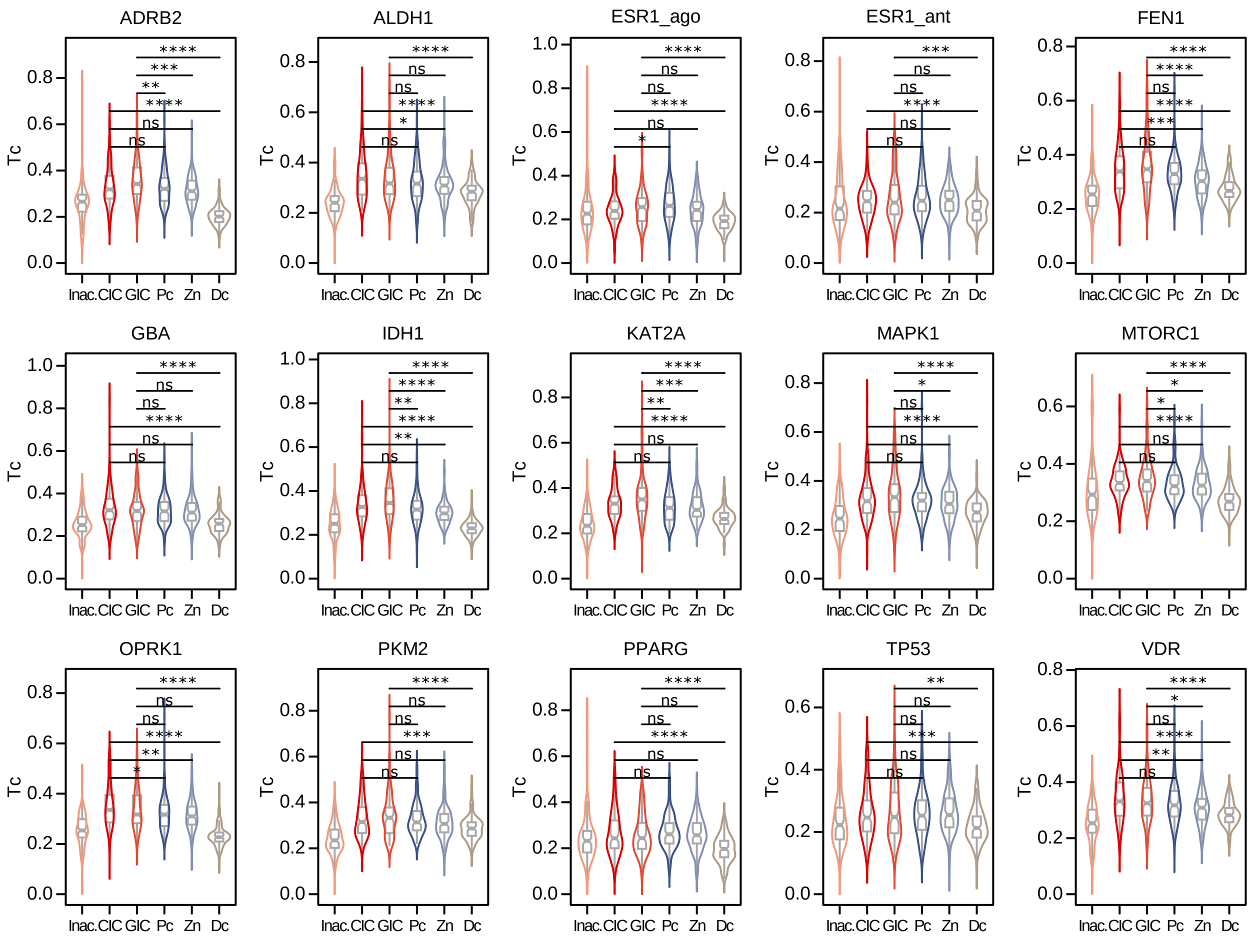

Supplementary Figure 12 | Chemical similarity analysis (LIT-PCBA). For each assay endpoint in the LIT-PCBA benchmark dataset, we compared the structural similarity between verified inactive compounds (Inac.) and compounds from InertDB (CIC and GIC subsets), PubChem (Pc), ZINC (Zn), and DeepCoy-generated decoys (Dc). Structural similarity was quantified using the Tanimoto coefficient (Tc), where for verified inactive compounds, the nearest neighbor Tc within the set was calculated. For InertDB, Pc, Zn, and Dc, Tc values were computed by sampling 100 compounds per set and determining their nearest neighbor Tc to verified inactive compounds. Statistical significance was assessed using the Wilcoxon test: *P < 0.05, **P < 0.01, ***P < 0.001, ****P < 0.0001, and ns (not significant).

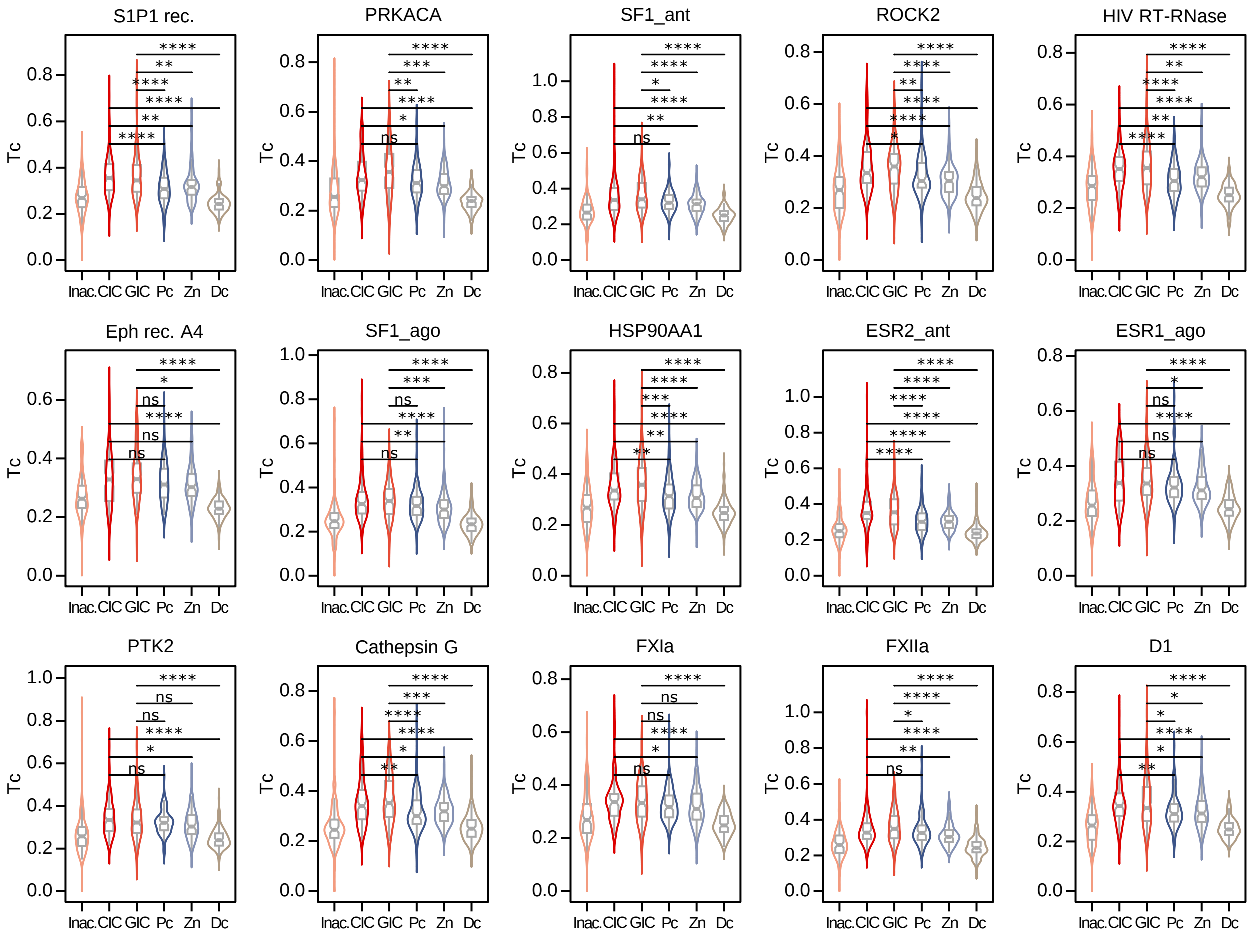

Supplementary Figure 13 | Chemical similarity analysis (MUV). For each assay endpoint in the MUV benchmark dataset, we compared the structural similarity between verified inactive compounds (Inac.) and compounds from InertDB (CIC and GIC subsets), PubChem (Pc), ZINC (Zn), and DeepCoy-generated decoys (Dc). Structural similarity was quantified using the Tanimoto coefficient (Tc), where for verified inactive compounds, the nearest neighbor Tc within the set was calculated. For InertDB, Pc, Zn, and Dc, Tc values were computed by sampling 100 compounds per set and determining their nearest neighbor Tc to verified inactive compounds. Statistical significance was assessed using the Wilcoxon test: *$P < 0.05$, **$P < 0.01$, ***$P < 0.001$, ****$P < 0.0001$, and ns (not significant).

Source	Assay name	Gene symbol	Target class	PubChem AID	Mode of interaction	# active	# inactive	Description
LIT-PCBA	ADRB2	ADRB2	GPCR	492947	Agonist	17	311748	Beta2 adrenergic receptor
	ALDH1	ALDH1A1	Enzyme (Dehydrogenase)	1030	Inhibitor	5363	101874	Aldehyde dehydrogenase 1
	ESR_ago	ESR1	NR	743075	Agonist	13	4378	Estrogen receptor alpha
	ESR_antago	ESR1	NR	743080	Antagonist	88	3820	Estrogen receptor alpha
	FEN1	FEN1	Enzyme	588795	Inhibitor	360	350718	Flap endonuclease 1
	GBA	GBA1	Enzyme	2101	Inhibitor	163	291241	Glucocerebrosidase
	IDH1	IDH1	Enzyme (Dehydrogenase)	602179	Inhibitor	39	358757	Isocitrate dehydrogenase
	KAT2A	KAT2A	Enzyme	504327	Inhibitor	194	342729	Histone acetyltransferase KAT2A
	MAPK1	MAPK1	Enzyme (Kinase)	995	Inhibitor	308	61567	Mitogen-activated protein kinase 1
	MTORC1	MTOR	Enzyme (Kinase)	493208	Inhibitor	97	32972	Mechanistic target of rapamycin
	OPRK1	OPRK1	GPCR	1777	Agonist	24	269475	Kappa opioid receptor
	PKM2	PKM	Enzyme	1631	Inhibitor	546	244679	Pyruvate kinase muscle isoform 2
	PPARG	PPARG	NR	743094	Inhibitor	24	4071	Peroxisome proliferator-activated receptor gamma
	TP53	TP53	Tumor suppressor	651631	Inhibitor	64	3345	Cellular tumor antigen p53
	VDR	VDR	NR	504847	Antagonist	655	262648	Vitamin D receptor
MUV	S1P1 rec.	S1PR1	GPCR	466	Agonist	223	55395	Sphingosine 1-phosphate receptor 1
	PKA	PRKACA	Enzyme (Kinase)	548	Inhibitor	62	64814	Protein kinase A catalytic subunit
	SF1	NR5A1	NR	600	Antagonist	213	64550	Steroidogenic factor 1
	Rho-Kinase2	ROCK2	Enzyme (Kinase)	644	Inhibitor	67	59576	Rho associated coiled-coil containing protein kinase 2
	HIV RT-RNase		RT-RNase (HIV)	652	Inhibitor	370	63969	HIV-1 reverse transcriptase
	Eph rec. A4	Epha4 (M. musculus)	RTK	689	Inhibitor	80	61480	EPH receptor A4
	SF1	NR5A1	NR	692	Agonist	75	63683	Steroidogenic factor 1
	HSP 90	HSP90AA1	HSP	712	Inhibitor	91	63481	Heat Shock Protein 90 Alpha Family Class A Member 1
	ER-a-coact. bind.	ESR1	NR	713	Antagonist	221	84656	Estrogen receptor alpha; ***Note***: DeepCoy failed.
	ER-β-coact. bind.	ESR2	NR	733	Antagonist	194	84984	Estrogen receptor beta
	ER-a-coact. bind.	ESR1	NR	737	Agonist	64	84947	Estrogen receptor alpha
	FAK	PTK2	Enzyme (Kinase)	810	Inhibitor	110	96070	Focal adhesion-associated protein kinase
	Cathepsin G	CTSG	Enzyme (Protease)	832	Inhibitor	65	62007	Cathepsin G
	FXIa	F11	Enzyme (Protease)	846	Inhibitor	70	218421	Factor XIa
	FXIIa	F12	Enzyme (Protease)	852	Inhibitor	99	216795	Factor IIa
	D1 rec.	DRD1	GPCR	858	Allosteric modulator	226	54292	Dopamine receptor D1
	M1 rec.	Chrm1 (R. norvegicus)	GPCR	859	Allosteric modulator	231	61477	Muscarinic acetylcholine receptor M1; ***Note***: DeepCoy failed.

Supplementary Table 1 | Description of LIT-PCBA and MUV datasets used for validation study of InertDB. The benchmark utilized two datasets: LIT-PCBA, containing information on active and inactive compounds for 15 assays, and MUV, covering 17 assays. For non-human protein targets, species information was indicated alongside the gene symbol. ***Note***: Two targets in the MUV dataset were excluded from the validation study for DeepCoy due to failure in decoy generation.
